# Intraductal tubulopapillary neoplasm (ITPN) of the pancreas associated with an invasive component: a case report with review of the literature

**DOI:** 10.1186/s12957-017-1267-4

**Published:** 2017-11-16

**Authors:** Stefanie Kuscher, Hartmut Steinle, Afschin Soleiman, Dietmar Öfner, Stefan Schneeberger, Georg Oberhuber

**Affiliations:** 1 Department of Visceral, Transplant and Thoracic Surgery, Center of Operative Medicine, Medical University of Innsbruck, Innsbruck, Austria; 2 Department of Internal Medicine I, Gastroenterology and Hepatology, Medical University of Innsbruck, Innsbruck, Austria; 3 Pathology Department of the General Hospital of Innsbruck, Innsbruck, Austria

**Keywords:** Intraductal, Pancreas, Neoplasia, Carcinoma, Pancreaticoduodenectomy

## Abstract

**Background:**

Intraductal tubulopapillary neoplasm (ITPN) depicts a distinct entity in the subgroup of premalignant epithelial tumors of the pancreas. Although the histomorphological and immunophenotypical characterization of ITPN has been described by several authors in terms of report of case series in the past, the rarity of that tumor subtype and similarity to other entities still makes identification of ITPN a challenge for radiologists and pathologists. To date, little is known about tubulopapillary carcinoma that can evolve from ITPN.

**Case presentation:**

In the present work, we analyze one case of ITPN associated with an invasive component and discuss the results involving the current literature. Collected patient data included medical history, clinical symptoms, laboratory tests, radiological imaging, reports of interventions and operation, and histopathological and immunohistochemical examinations. The patient initially presented with acute pancreatitis. A solid tumor obstructing the main pancreatic duct and sticking out of the papilla of Vater was detected and caught via endoscopic intervention. Histopathological examination of the specimen revealed mainly tubular growth pattern with back to back tubular glands. Immunohistochemically, the tumor was strongly positive for keratin 7 (CK7) and pankeratin AE1/AE3, and alpha 1 antichymotrypsin; negative for synaptophysin and chromogranin A, CDx2, CK20, S100, carcinoembryonic antigen (CEA), MUC 2, MUC5AC, and somatostatin; and in part positive for CA19-9. Extended pancreatoduodenectomy was performed, the final diagnosis was tubulopapillary carcinoma grown in an ITPN.

**Conclusion:**

The identification of an ITPN of the pancreas can be a challenging task. Endoscopic retrograde cholangiopancreaticography is an excellent tool to directly see and indirectly visualize the intraductal solid tumor and to take a biopsy for histopathological evaluation at the same time. Together with a thorough immunohistochemical workup, differential diagnoses can be ruled out quickly. To date, reports of ITPN are rare and little is known about the potential for malignant transformation and the prognosis of tubulopapillary carcinoma grown from an ITPN. Radical surgical resection following oncologic criteria is recommended; however, more data will be needed to assess an adequate treatment and follow-up standard.

## Background

Intraductal tubulopapillary neoplasm was first recognized by Japanese investigators [1]. In 2002, the Japan Pancreas Society proposed the name intraductal tubular carcinoma which was in 2009 changed to intraductal tubulopapillary neoplasm. In 2010, the World Health Organization (WHO) recognized intraductal tubulopapillary neoplasms (ITPNs) of the pancreas as a distinct entity which was included in the subgroup of premalignant epithelial tumors of the pancreas. It was defined as intraductal, grossly visible, tubule-forming epithelial neoplasm with high-grade dysplasia and ductal differentiation without overt production of mucin.

In the current edition of the WHO tumor classification, only ITPN and intraductal pancreatic mucinous neoplasm (IPMN) are included in the category intraductal neoplasms. Other entities which may histologically mimic ITPN and may show intraductal growth such as acinar cell carcinoma and pancreatic neuroendocrine tumor are not included in this category, as they show a nonductal differentiation.

ITPN is a rare tumor and accounts for less than 1% of exocrine pancreatic neoplasms. It usually appears as a solid mass obstructing the main pancreatic duct thereby causing upstream duct dilation. Due to its malignant potential, ITPN is usually treated by radical surgical resection following oncological criteria in a curative approach [2]. Pylorus-preserving pancreaticoduodenectomy is one of the most frequently performed surgical procedures for that purpose, as ITPNs are known to be located in the head of the pancreas in up to 50%.

Whereas histopathological and immunomorphological characteristics are well described, little is known about surgical outcome and prognosis of ITPN to date.

## Case presentation

The 73-year-old male patient initially presented in the emergency room with nausea and persistent abdominal pain in the upper right quadrant for 1 day. He reported to feel weak and exhausted for a few weeks already. A weight loss of 5 kg in 2 months was attributed to a special diet; the patient did not record loss of appetite. Night sweating was negated. The medical history did not reveal a malignant or benign disease of the gastrointestinal tract. A routinely performed screening colonoscopy 3 years ago showed normal findings.

Elevation of serum amylase and lipase, liver transaminases, and bilirubin as well as the patient’s clinical presentation indicated acute pancreatitis (Table 1).

**Table 1:** Blood test results at the patient's first presentation

Day of initial presentation	Pancreas alpha-amylase (13–53 U/l)	Lipase (13–60 U/l)	ALT (10–50 U/l)	AST (10–50 U/l)	gGT (10–71 U/l)	AP (40–130 U/l)	Bilirubin (0–1.28 mg/dl)	CRP (0–0.5 mg/dl)	WBC (4–10 G/l)
Day 1	266	374	229	339	194	116	3.05	0.94	7.3
Day 2	75	79	172	128	192	98	4.73	10.96	11.3
Day 3	246	390	119	74	172	109	3.37	11.72	5.1
Day 6	53	121	160	123	219	152	1.05	3.67	3.6
Day 20	46	97	36	23	91	105	0.98	0.15	3.0

Serum values of amylase, lipase, AST, ALT, gGT, AP, bilirubin, CRP, and WBC were elevated at the patient’s initial presentation, pointing to biliary pancreatitis. After ERCP and pancreatic duct stenting on day 2, the enzymes initially elevated but then decreased rapidly towards normal values

**Fig. 1:**
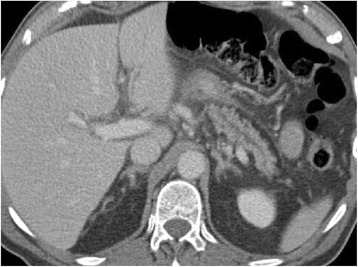
CT of the abdomen (portalvenous phase) at the initial presentation shows a dilated pancreatic duct without evidence of an obstructive process or cholelithiasis

Contrast-enhanced computed tomography (CT) of the abdomen confirmed the diagnosis of pancreatitis, showing edematous peripancreatic fat, minimal amounts of ascites, accentuated peripancreatic lymph nodes, a dilated pancreatic duct in the tail and body of the pancreas, and slightly dilated intra- and extrahepatic bile ducts. There were no clear signs of a tumor or stenosing process in the pancreas and bile ducts at that time (Fig. 1).

**Fig. 2:**
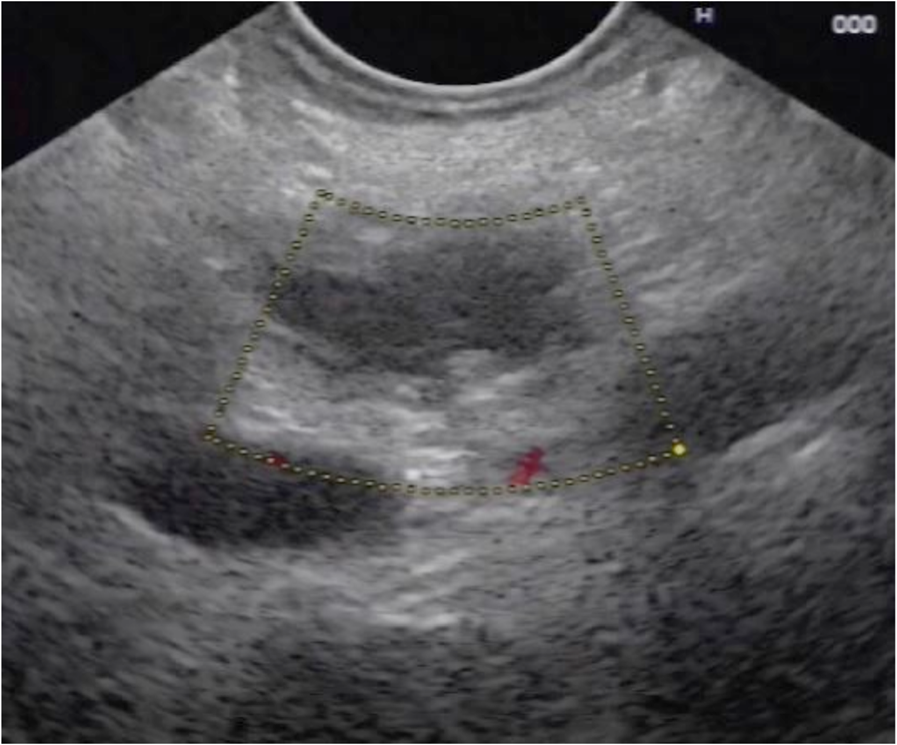
Transgastric endosonography reveals a dilation of the pancreatic duct to a diameter of approximately 7 mm

**Fig. 3:**
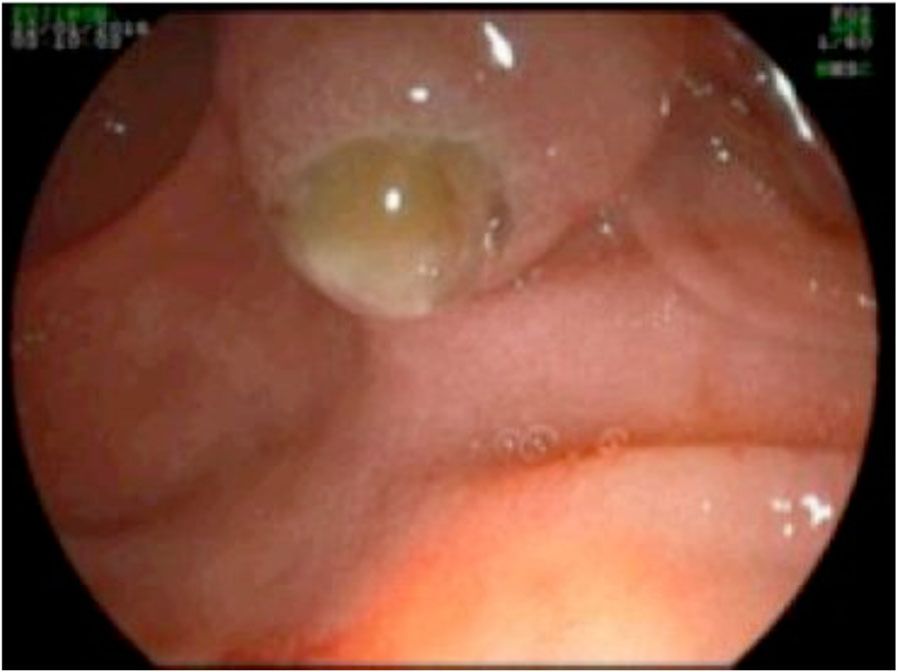
A snapshot on ERCP shows a thrombus-like formation sticking out of the papilla of Vater

**Fig. 4:**
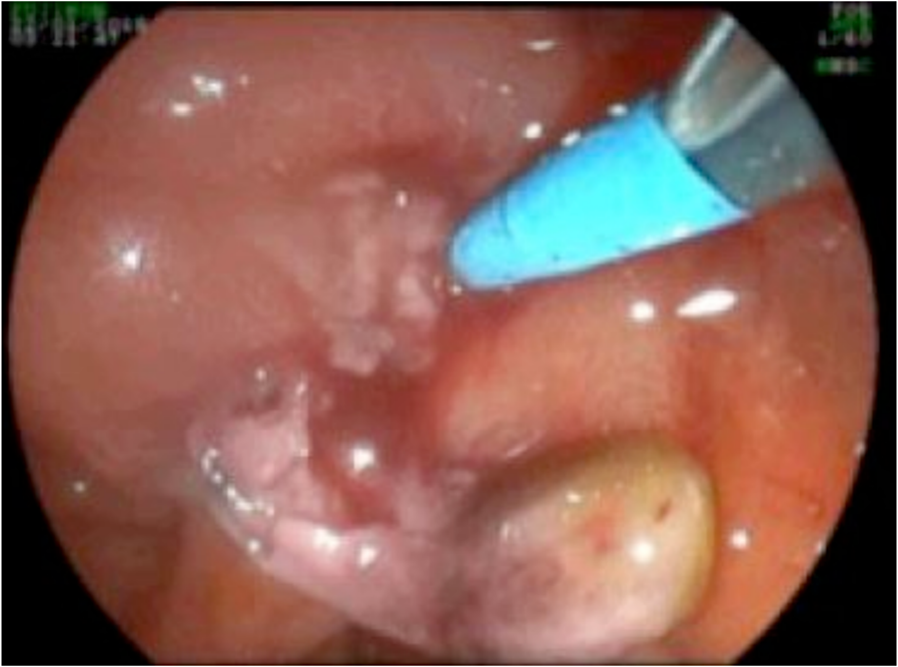
After slight manipulation at the papilla, a tumor nodule drops out of the duct and can be collected via endoscopy

**Fig. 5:**
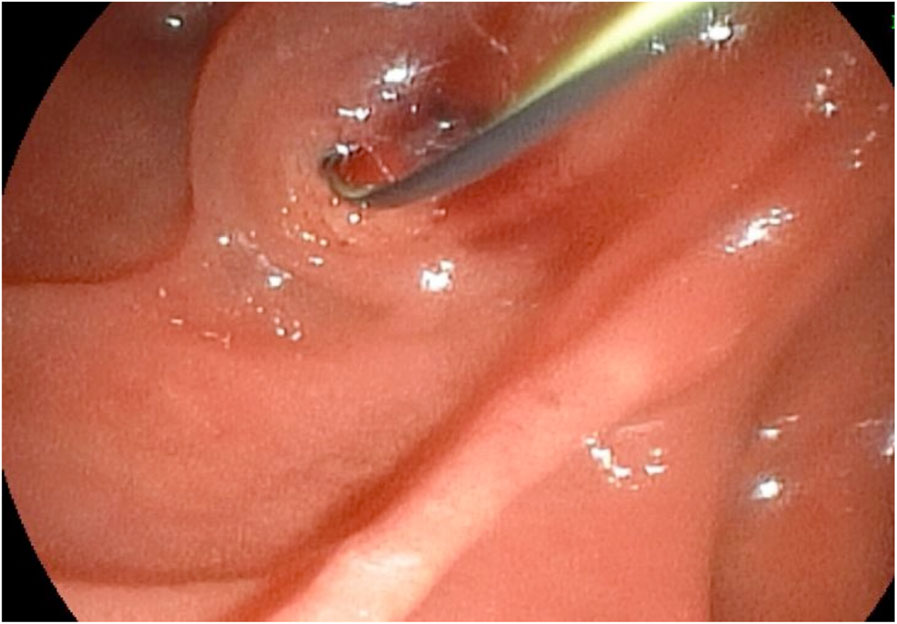
After the specimen has been removed, intubation of the papilla can be performed for pancreatography

**Fig. 6:**
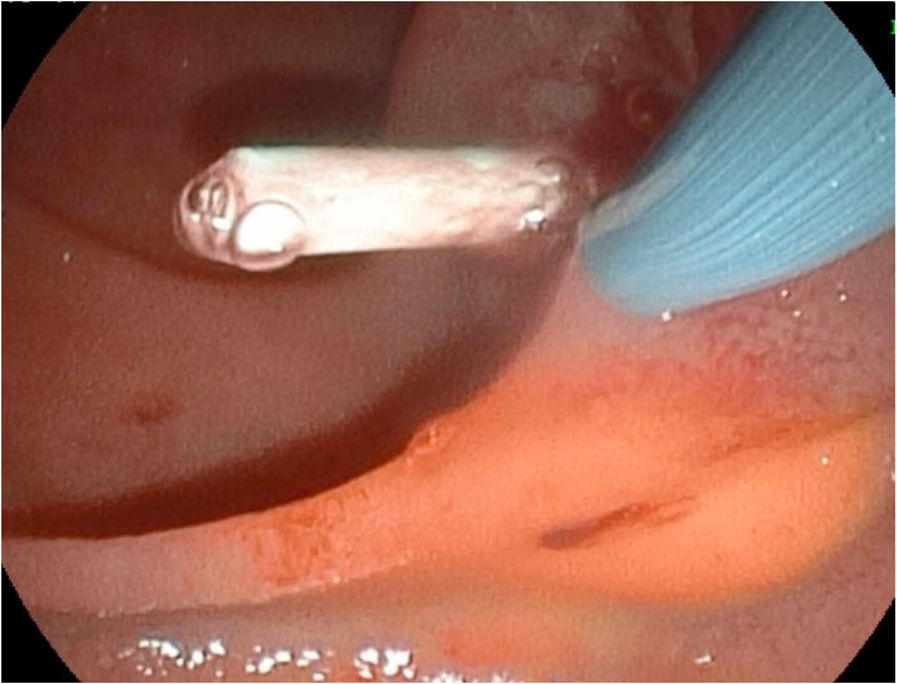
A 7-French plastic stent is placed into the congested distal pancreatic duct in order to drain the dilated duct of the pancreatic body and tail

Conservative treatment and further evaluation including endosonography and endoscopic retrograde cholangiopancreaticography (ERCP) were initiated. Endosonography revealed an enlarged pancreatic head with inhomogenous parenchyma, compromising the distal bile duct, but without signs of mechanic cholestasis or cholelithiasis. The pancreatic duct was dilated to 7 mm (Fig. 2), with a vaguely discernible configuration of the duct wall, being suspicious of main-duct-intraductal pancreatic mucinous neoplasm (IPMN). ERCP showed an atypical tissue fragment sticking out of the swollen orifice of the papilla of Vater, considered either to be a thrombus, sludge, or neoplasia (Fig. 3). With minimal manipulation, the specimen dropped into the duodenum and could be collected for histopathological examination (Fig. 4). Bile duct intubation was unsuccessful. Pancreatography revealed congestion of the distal duct, a protective plastic stent was therefore inserted, and an aspirate probe was taken for cytological evaluation (Figs. 5 and 6). During the procedure, the examiner did not notice mucin content.

Histopathological and immunohistochemical workup (antibodies listed in Table 2) of the specimen collected via endoscopy yielded the unexpected diagnosis of a partly necrotic, highly differentiated pancreatic tumor, strongly positive for keratin 7 (CK7) and pankeratin AE1/AE3, and alpha 1 antichymotrypsin; negative for synaptophysin and chromogranin A, CDx2, CK20, S100, MUC 2, MUC5AC, carcinoembryonic antigen (CEA), and somatostatin; and in part positive for CA19-9 (20% of cells positive). A proliferation rate of 20% was revealed by Ki-67 staining. CK7 expression in combination with the histological feature of a tumor with back to back tubular glands was indicative of intraductal tubulopapillary neoplasia. In the absence of desmoplastic stroma reaction, classic pancreatobiliary carcinoma was considered unlikely. A neuroendocrine tumor was excluded due to the negative immunohistological reaction to neuroendocrine markers.

**Table 2:** Antibodies for immunohistochemistry

Target antigen	Provider	Article number	Dilution	Clone
CK AE1/AE3	DAKO	GA053	RTU	AE1/AE3
Antichymotrypsin	Menarini	44421	1:80	Polyclonal
Synaptophysin	DAKO	IR660	RTU	DAK-SYNAP
Chromogranin A	DAKO	M0869	1:200	DAK-A3
CDx2	DAKO	GA080	RTU	DAK-CDX2
CK20	DAKO	GA777	RTU	KS20.8
CK7	DAKO	GA619	RTU	OV-TL 12/30
CK19	DAKO	GA615	RTU	RCK108
S100	DAKO	GA504	RTU	Polyclonal
CEA	DAKO	GA622	RTU	II-7
Somatostatin	DAKO	A0566	1.200	Polyclonal
CA 19.9	DAKO	M3517	1:70	1116-NS-19-9
Ki-67	DAKO	GA626	RTU	MIB-1
MUC2	DAKO	IR658	RTU	CCP58
MUC5AC	DAKO	IR661	RTU	CLH2

The antibodies used for the specific molecular characterization of ITPN and distinction from other entities are listed above

*RTU* ready to use

Cytological evaluation of the pancreatic duct aspirate was performed in an extern laboratory. The probe did not contain any cells or mucin and was D-PAS negative. KRAS mutation was not detected. The patient was checked for abnormality of serum immunoglobulins, in particular IgG4 and tumor markers, but showed no relevant increase of alpha-1-fetoprotein, CEA, cancer antigen (CA) 19-9, CA72-4, CA125, IgA, IgM, or IgE. Only IgG subclass 2 was unspecifically elevated.

Eighteen days after the initial endoscopic intervention with pancreatic stenting, re-endoscopy with stent removal and follow-up endosonography were performed. Pancreatic duct diameter had normalized. A highly suspect 11-mm hypoechoic vascularized lesion with sharp limitation, contacting but not compromising the pancreatic duct, was detected in the pancreatic body. In the inhomogenously structured pancreatic head, pancreatitis seemed to have regressed, but a hypoechoic formation without a clear border was seen, suspicious of pancreatic carcinoma. The distal common bile duct was compressed in the pancreatic head with a slight prestenotic dilatation.

**Fig. 7:**
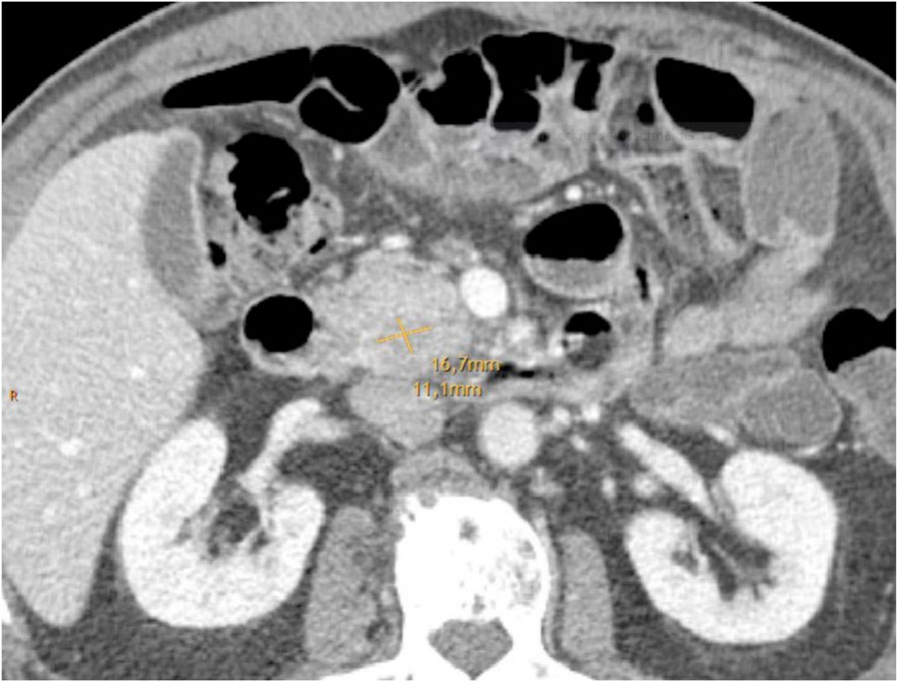
Contrast-enhanced CT in portal venous phase shows the hypodense lesion in the pancreatic head

Staging CT of the chest, abdomen, and pelvis confirmed the presence of a suspect hypodense lesion located in the head of the pancreas (Fig. 7), and accentuated peripancreatic, mesenteric, and parailiacal lymph nodes with a maximum diameter of 8 mm were described. There was no indication of metastatic tumor spread. Magnetic resonance cholangiopancreaticography (MRCP) was not performed.

Following interdisciplinary consensus in the hepato-pancreato-biliary tumor conference, oncologic extended pylorus-preserving pancreaticoduodenectomy (PPPD) was planned. After another short episode of a mild pancreatitis and thorough anaesthesiological evaluation prior to surgery, extended PPPD with pancreatogastric anastomosis was performed without complications on day 47 after initial admission to hospital. Intraoperative immediate frozen sections of the resection margins of the common bile duct and pancreas were free of tumor cells. Intraoperative sonography of the liver showed no sign of hepatic tumor spread.

In the formalin-fixed surgical specimen, macroscopy revealed a narrowed pancreatic duct in a distance of 1 cm from the papilla, occluded by a drop-shaped 8-mm tumor that appeared to leave the duct wall intact for the most part. The organ parenchyma appeared partly fibrous with regular acini in the uncinate process.

**Fig. 8:**
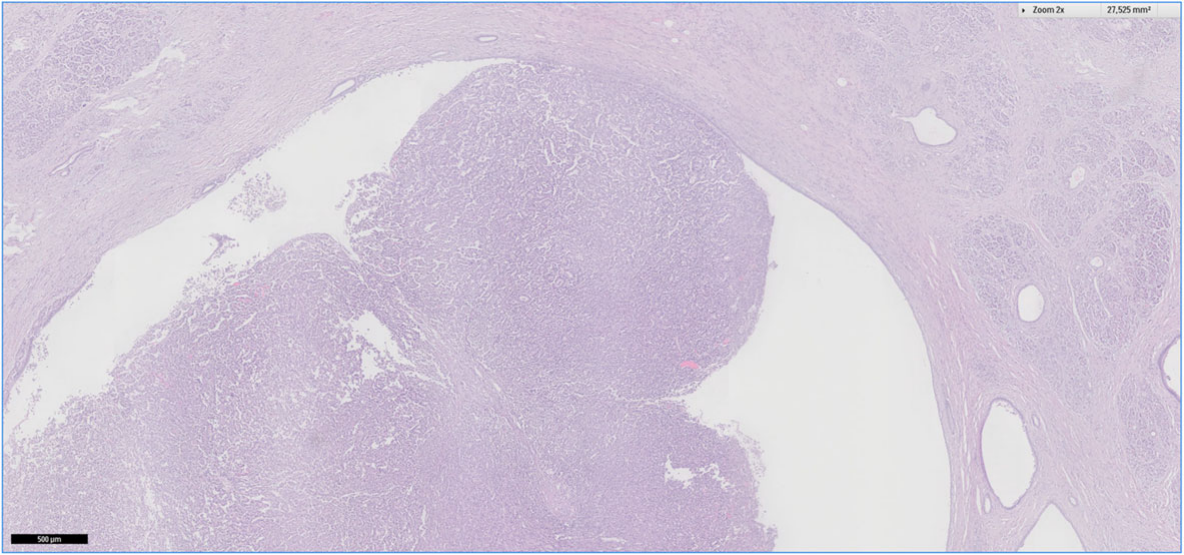
This histological slide depicts an intraductal growing area of the tumor in the main pancreatic duct, which was initially observed at endoscopy. A large percentage of the duct’s circumference is lined by normal appearing epithelial cells. Hematoxylin and eosin, ×100

**Fig. 9:**
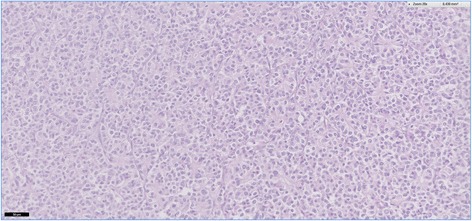
A higher magnification of the intraductal tumor with back to back glands. Hematoxylin and eosin, ×300

**Fig. 10:**
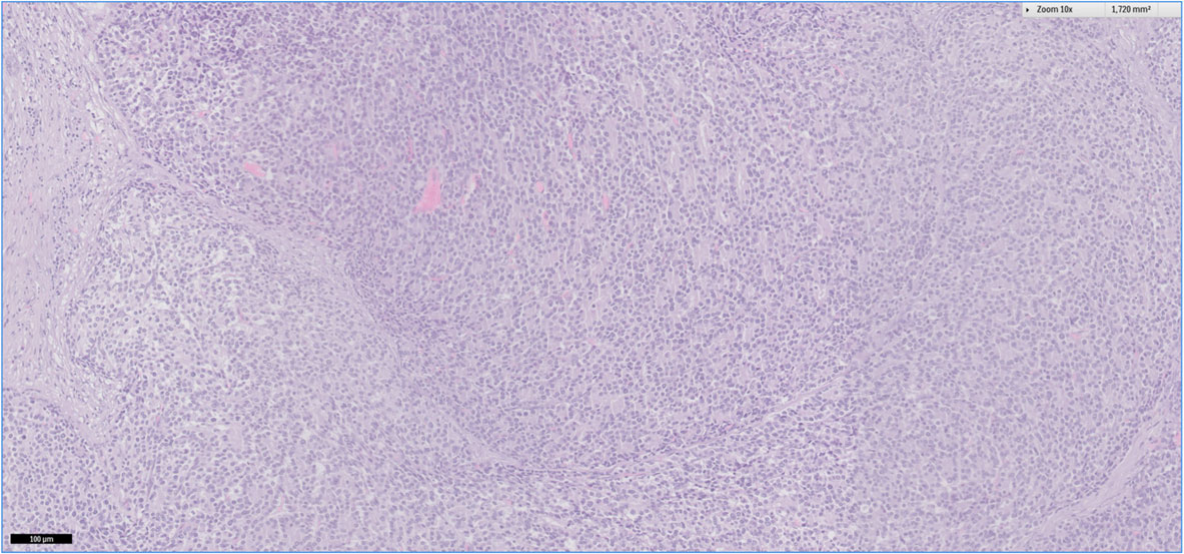
In this area, the neoplasia was characterized by a nodular tumor growth. Although the appearance is suggestive of invasive disease, intraductal growth only cannot be ruled out. Hematoxylin and eosin, ×200

Histopathological examination of HE-stained sections taken form most parts of the resection specimen revealed an intraductal lumen-occluding lesion with a mainly tubular growth pattern with back to back tubular glands (Figs. 8 and 9). In areas with intraductal growth, a remainder of the original ductal epithelium was observed in some ducts. Nodular tumor cell aggregates showing the same histomorphological appearance were seen extraductally (Fig. 10). Those nodules infiltrated the normal pancreatic tissue, reaching the organ margins without invasion of the peripancreatic fatty tissue. The remaining parenchyma was fibrotic with signs of chronic pancreatitis.

**Fig. 11:**
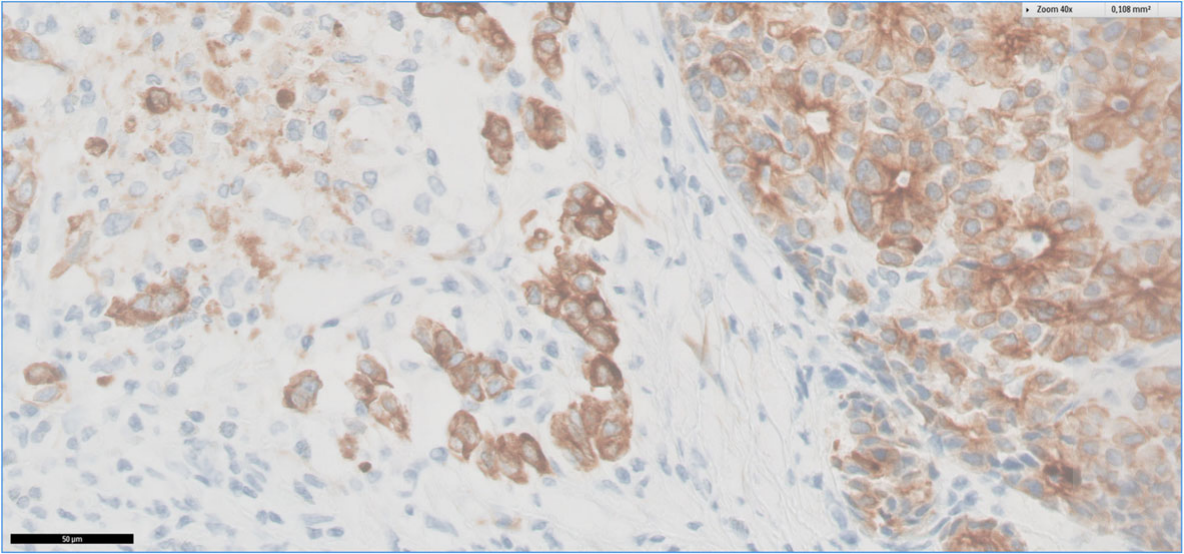
Keratin 7-positive tumor cells are labeled with a brown coloration. In the center of the slide, there are single tumor cells indicating invasive disease. Anti-CK7, DAB

Immunohistology revealed the same findings as compared to the biopsy specimen including a strong expression of keratin 7 by all tumor cells (Fig. 11). Neuroendocrine markers (synaptophysin, chromogranin A) were negative as was a trypsin stain. Keratin 19 was positive in approximately 10% of tumor cells.

Definite histopathological diagnosis was a tubulopapillary carcinoma of the pancreatic head grown from an intraductal tubulopapillary neoplasm with a maximal tumor diameter of 28 mm.

According to the Union for International Cancer Control (UICC) 8th edition TNM staging for malignant tumors, the tumor was classified as pT2, pN0 (0/25), L0, V0, Pn0, R0 (wide), which refers to a stage IB. The tumor was considered low-grade (G1) [3].

The patient could be discharged from hospital on postoperative day (POD) 13 after an uneventful postoperative course. Six weeks after the operation, on POD 43, the patient was readmitted for 1 day due to unspecific upper abdominal pain and vertigo. Clinical examination showed a distended abdomen, but no signs of peritonitis. Abdominal sonography revealed slightly reduced bowel movements. There were no signs of cholestasis or fluid collections seen on ultrasound examination. Blood test showed normal levels of serum pancreatic and liver enzymes as well as normal inflammation parameters (Table 3). Regular postoperative findings were recorded on abdominal CT. After fast recovery under conservative treatment with bland diet and analgetics, the patient could be discharged in good general condition.

**Table 3:** Postoperative blood test results

Postoperative day (POD)	Pancreas alpha-amylase (13-53 U/l)	Lipase (13–60 U/l)	ALT (10–50 U/l)	AST (10–50 U/l)	gGT (10–71 U/l)	AP (40–130 U/l)	Bilirubin (0–1.28 mg/dl)	CRP (0–0.5 mg/dl)	WBC (4–10 G/l)
POD 12	6	6	48	25	87	104	0.26	2.17	5.9
POD 43	25	29	31	23	30	95	0.39	0.07	6.0
POD 102	–	–	23	25	47	114	0.33	0.35	3.6
POD 193	–	–	24	21	99	168	0.45	0.74	3.5

Laboratory findings at different time points are shown in Table 2. Liver and pancreatic enzymes and parameters of cholestasis and inflammation were normal when the patient was readmitted on POD 43 due to abdominal discomfort

Due to the rarity of that specific tumor entity and data postulating lower malignancy than in ductal adenocarcinoma, the interdisciplinary tumor conference decided for short interval follow-up (3 months) without adjuvant chemotherapy.

The patient recently had the second follow-up in our hepatopancreatobiliary outpatient clinic 6 months after pancreatic resection. Physical examination as well as blood tests and abdominal sonography showed normal findings, and the patient presented clinically well under pharmacological pancreatic enzyme substitution. The next follow-up appointment after three more months is planned to include an abdominal CT.

## Discussion and conclusion

Intraductal tubulopapillary neoplasm (ITPN) depicts a rare subgroup of intraductal epithelial neoplasms of the pancreas. Yamaguchi et al. were the first ones to collect and describe 10 cases of that previously undefined type of tumor [1]. In the 4th edition of WHO tumor classification in 2010, ITPN was then determined one distinct entity of premalignant epithelial tumors of the pancreas besides IPMN, PanIN grade 3, and mucinous cystic neoplasm.

ITPN most commonly shows the morphological appearance of a solid nodular tumor arising in the main pancreatic duct and obstructing the ducts with subsequent upstream dilatation [1]. Consistent with that, our patient showed a polypous lesion sticking out of the papilla of Vater, causing pancreatic duct obstruction and dilatation and reactive pancreatitis. Due to unspecific symptoms such as discomfort, abdominal pain, and weight loss, the lesion is often only detected incidentally on abdominal imaging examination [4]. A discrimination between ITPN and IPMN based on imaging findings in abdominal sonography, endosonography, CT and magnetic resonance imaging (MRI) techniques, and endoscopic retrograde cholangiopancreaticography (ERCP) can be challenging, as both entities show similar properties [2, 5]. In 2012, Motosugi and colleagues published a synopsis on radiomorphological features of 11 histologically confirmed ITPN in 10 patients, 9 of whom belonged to the study population of Yamaguchi’s first report of ITPN [1, 6]. On contrast-enhanced CT, low density of the ITPN lesion compared to the adjacent pancreatic parenchyma was detected in arterial, portal venous, and delayed phase. Agreeing with that, our patient’s tumor presented as hypodense formation in the pancreatic head on contrast-enhanced CT (pancreatic parenchyma phase). In six out of seven patients that underwent MRI, signal intensity of ITPN was low in T1-weighted MRI and high in T2-weighted MRI. Moreover, Motosugi postulated intraductal tumor growth could be detected best on endoscopic or transabdominal ultrasonography [6]. A two-tone duct sign was frequently seen on MRCP and ERCP, meaning a pancreatic duct that appears in two colors, one of the dilated pancreatic duct and one of the intraductal tumor. Once the mass fills the duct lumen, an abrupt disruption is seen. If the tumor does not obstruct the duct and hence is surrounded by pancreatic fluid, a cork of wine bottle sign can be described. However, the retrospective study design and non-uniform radiologic examination of patients in multiple institutions, as well as the lacking comparison to imaging of other pancreatic neoplasms, clearly limit the conclusions to be taken from this study [6].

Typical features of ITPN on histopathological examination are a tubulopapillary growth pattern, high-grade dysplasia, necrotic foci, and a lack of visible intracellular mucin, which distinguish ITPN from IPMN [1]. In contrast, acinar cell carcinoma, neuroendocrine tumor, and in biopsy specimens also, solid pseudopapillary neoplasm, may show similar histopathologic features and cannot be distinguished in conventional histology. However, a differing immunohistological pattern allows the distinction of those entities [7]. In our case, the lack of the neuroendocrine markers synaptophysin and chromogranin ruled out a pancreatic neuroendocrine tumor. The strong expression of CK7 in combination with negative trypsin stain militated against acinar cell carcinoma. The histomorphological features were not compatible with a solid pseudopapillary neoplasm.

Regarding immunohistochemical appearance, ITPN was reported lacking gastroenteric differentiation, typically seen in IPMN, reflected by the absence of MUC2 (and of CDX2) and MUC5AC expression. Instead, signs of pancreatic duct differentiation are revealed by expression of CK7 and/or CK19, as well as MUC1 and MUC6 [2, 4, 5, 8–12]. A large series of ITPN (*n* = 33) recently reported by Basturk et al. focused on a thorough clinicopathological characterization of those uncommon pancreatic lesions. Data from five high-volume centers of surgical pathology were collected and reviewed for that purpose. Besides the aforementioned characteristics, the authors noticed CAM5.2 labeling of all ITPNs, as a proof of epithelial tissue origin, and the absence of MUC2 and MUC5AC [4].

Date et al. screened the literature for reports on ITPN in 2016 and retrospectively analyzed a total of 58 cases, including Yamaguchi’s first cases [2]. In their summary of immunohistochemical features of ITPN, they also reported that CK7 and CK19 were typically positive while trypsin, MUC2, and MUC5AC were negative. The absence of the neuroendocrine markers synaptophysin and chromogranin A, another suggestive fact on the diagnostic pathway, was recorded in their own ITPN case. In 2015, Muraki et al. published their workup of an intraductal neoplasm showing typical clinical and histomorphological features of an ITPN but exhibiting MUC5AC expression and hence impeding the discrimination between ITPN and pancreatobiliary-type IPMN [13]. The authors appealed on a more precise classification of those neoplastic lesions. Another Japanese group emphasized the importance of a thorough characterization and the publication of each precisely described ITPN case in order to gain more detailed information about that rare entity [14]. They recently published their own case of an ITPN that showed the classic immunomorphological phenotype with expression of CK7 and CK19, and negativity for MUC2 and MUC5AC. The lesion occupied the entire pancreas with intraductal growth and infiltration of the main pancreatic duct, and total pancreatectomy with splenectomy was required [14].

MUC5AC has been found to be expressed commonly by pancreatic ductal adenocarcinoma (PDAC) and, less frequently, by precursor lesions such as IPMN [15–17]. As mentioned above, ITPN typically does not express MUC5AC. Some authors noted that MUC5AC expression on pancreatic ductal adenocarcinoma (PDAC) comes along with better outcome, meaning higher survival rates, compared to MUC5AC-negative PDAC [18, 19]. On the other hand, it has been shown in experimental models that MUC5AC aids cancer cells escape from the immune system and is therefore associated with poor outcome [20]. Investigations on the mucine profile of pancreatic neoplasms are being performed to explore the value of MUC5AC expression and other mucines as prognostic markers and potential therapeutic targets [15, 21–23].

ITPN show genetic patterns that are different from those seen in IPMN, PanIN, and pancreatic ductal adenocarcinoma, specifically an absence of KRAS and BRAF mutations [1, 2, 4, 8, 11]. Yamaguchi et al. worked up 14 ITPNs and 15 IPMNs of the gastric-type, pyloric gland variant (IPMN-PG), in order to explore and compare the two entities’ molecular genetic fingerprints. They found KRAS mutations in only 1 ITPN but in 12 IPMN-PGs. BRAF mutation was detected in 1 ITPN but in none of the IPMN-PGs, and GNAS mutation was evident in none of the 14 ITPNs but in 9 of 15 IPMN-PGs [24]. The prevalence of KRAS and GNAS mutations in IPMNs as well as the typical absence of KRAS mutation in ITPNs was described by other groups as well [25]. Recently, Basturk et al. published their thorough workup including genomic analyses of 22 ITPN cases. The authors postulate that the distinct genetic characteristics they could show in ITPN and IPMN might serve as specific targets for innovative therapeutic means in the future [26].

Intraductal tubulopapillary neoplasms have also been reported to occur in the bile ducts [27, 28]. Nakagawa et al. found an intraductal neoplasia in an intrahepatic bile duct, showing similar histological and immunohistochemical features as ITPN of the pancreas [29]. Investigations on 20 bile duct ITPNs in 2015 revealed the high prevalence of an invasive carcinoma component (80%) but with a 5-year survival rate of 90% pointing to a less-aggressive malignancy compared to other epithelial carcinomas in that region [28].

Addressing the need for early detection and discrimination of different pancreatic lesions, Tajima et al. reflected the value of endoscopic ultrasound-guided fine needle aspiration cytology (EUS-FNAC) and described their observations in one case [30]. Three conspicuous findings were recorded on cytological evaluation: papillary cellular clusters with branching, tubules in contact with fibrovascular structures and cribriform structures. Loose cohesiveness of constituent cells was observed. Individual cells showed relatively uniformly enlarged nuclei with parachromatin clearing and distinct nucleoli. Morphological appearance and lack of cytoplasmic mucin lead to the suspected diagnosis of ITPN with a carcinoma component. After radical surgical resection, histopathological workup of the specimen revealed the same morphological patterns as seen in cytological examination and confirmed the diagnosis of ITPN [30]. EUS-FNAC was postulated a valuable diagnostic tool by other authors as well. The presence of thin fibrovascular structures surrounded by tubules and cribriform structures in ITPN are considered important cytological features to distinguish ITPN from IPMN [30–32]. More precisely, Aslan and colleagues emphasized the presence of highly cellular three-dimensional clusters showing complex branching, tubular and cribriform patterns, the absence of true papillary structures containing a fibrovascular core, and the absence of intracytoplasmic mucin, as well as uniformly enlarged nuclei with distinct nucleoli and scarcity of mitosis as typical features shown by ITPN [31]. Tajima et al. came to the conclusion that the typical three-dimensional clusters and tubules in contact with fibrovascular structures are detectable by FNAC and possibly by pancreatic duct brushing cytology, but not in cytological examination of pancreatic fluid [30].

Unspecific symptoms such as discomfort, abdominal pain, weight loss, and jaundice due to pancreatic duct obstruction may be recorded in ITPN patients [11]. In their study population, Basturk et al. recorded no specific disease-related symptoms in 18 (54.5%) out of 33 patients.

In the study reported by Basturk et al., 45% of ITPN were located in the head of the pancreas, 32% in the body/tail, and 23% diffusely involved the organ [4], agreeing with Kölby’s observation of 52% of ITPN located in the pancreatic head, 17% in the body, 7% in the tail, 3% in both head and body, and 14% in the whole pancreas [11]. Rooney et al. reported pancreatic head and pylorus-preserving pancreatoduodenectomy to be the most commonly performed therapeutic procedure for ITPN in a curative attempt [9]. The patient we present also showed tumor growth in the pancreatic head and underwent extended pancreaticoduodenectomy following oncologic criteria. On the surgical specimen, maximum tumor diameter of 2.8 cm was measured. Basturk et al. quoted a median tumor size of 4.5 cm, and within the tumor mass, both cystic and solid regions were described. In their literature review and collection of 30 ITPN cases, Kölby et al. recorded a median tumor diameter of 3 cm.

A significant proportion of ITPN may become invasive [4]. The invasive component may be morphologically similar to the intraductal one or may be highly infiltrative resembling conventional ductal carcinoma. Whereas the ductal carcinoma like invasive tumor is easily recognized, this is not the case with the tumors showing a tubulopapillary pattern. Invasive parts of the ITPN form round aggregates that closely resemble areas with intraductal growth. Invasiveness can only be determined by carefully examining the borders of the aggregates, which may be slightly irregular. Also, invasive areas are always devoid of remaining duct cells, which are often found in intraductal growing parts of the tumor. Finally, the distribution of the aggregates, which are observed in areas devoid of ducts, aids in the diagnosis of invasiveness. In our case, the invasive component was exclusively similar to the intraductal one. This fact readily explains the findings in radiology and ultrasound, which were not considered specific for invasive ductal carcinoma of the pancreas.

In the study of Kölby et al., one patient died from multiple liver metastases after 7 months, two other patients had successful treatment for tumor recurrence 12 and 34 months postoperatively, and two patients required palliative care for recurrent disease with distant metastases after 18 and 24 months [11]. The authors pointed out that recurrence may have been caused by intraductal colonization after distal pancreatectomy. However, distal pancreatectomy may be saved in a considerable proportion of patients as demonstrated by the disease-free survival in others that underwent distal pancreatectomy.

In the study by Basturk et al., follow-up data of 22 patients were available. Among those, the 5-year survival rate was 100% in patients without an invasive component and 71% in patients with an invasive carcinoma. Two patients died of the disease at 23 and 41 months; one patient died of unrelated causes at 49 months. After a median follow-up period of 61.5 months, 12 patients were alive with disease, while 7 patients were disease-free after a median follow-up of 19 months [4]. When Date et al. collected published data from patients surgically treated for ITPN of the pancreas in order to determine outcome, they reported 37 cases in whom an overall postoperative 5-year survival rate of 80.7% was detected. However, the value of those results is clearly limited since the authors included cases from the era before diagnostic guidelines for ITPN were defined [2]. Anyway, several authors postulated a relatively favorable overall outcome after curative resection compared to conventional pancreatic ductal adenocarcinoma [4, 8, 9, 12].

Satisfying results have been shown after radical resection of pancreatic ITPN, compared to the more malignant course of PDAC. Following oncologic R0 resection, our patient did not show any sign of disease recurrence in the short postoperative period of 6 months. The interdisciplinary tumor board decided for initial 3-monthly follow-up in our specialized hepatopancreatobiliary surgical outpatient unit. Close clinical follow-up is recommended for early detection of disease recurrence [11].

To date, reports of ITPN with or without invasive carcinoma are rare and case series largely make recourse to earlier publications. In order to assess the long-term outcome of ITPN and to develop appropriate therapeutic strategies, more data will be needed in the future.

## Abbreviations

CEA: Carcinoembryonic antigen
CT: Computed tomography
ERCP: Endoscopic retrograde cholangiopancreaticography
EUS-FNAC: Endoscopic ultrasound-guided fine needle aspiration cytology
IPMN: Intraductal papillary mucinous neoplasia
ITPN: Intraductal tubulopapillary neoplasm
MRI: Magnetic resonance imaging
PanIN: Pancreatic intraepithelial neoplasia
PDAC: Pancreatic ductal adenocarcinoma
PPPD: Pylorus-preserving pancreaticoduodenectomy
WHO: World Health Organization
